# Junctional kyphosis: how can we detect and monitor it during growth?

**DOI:** 10.1186/s13013-016-0100-0

**Published:** 2016-10-17

**Authors:** Alessandra Negrini, Sabrina Donzelli, Laura Maserati, Fabio Zaina, Jorge H. Villafañe, Stefano Negrini

**Affiliations:** 1 ISICO (Italian Scientific Spine Institute), Milan, Italy; 2 University of Brescia, Brescia, Italy; 3 IRCCS Don Gnocchi, Milan, Italy

## Abstract

**Background:**

Despite its importance in affecting adult pain, and disability, there is a lack of universal criteria for the diagnosis and evaluation of thoraco-lumbar Junctional Kyphosis (JK) and a gold standard measurement and diagnostic system does not exist.

This study aims to verify the sensibility and specificity of clinical, and Formetric surface topography (FST) data in identifying Junctional Kyphosis in respect to the radiographical standard references.

**Methods:**

Design: This is a cross sectional study from a prospective database started in March 2003.

Participants: 38 subjects.

Inclusion criteria: Patients selected by age according to Risser score 1, at first visit with lateral x-rays and FST. Diagnostic test used to detect JK:

- FST criteria: level of thoraco-lumbar inflexion point in percentage compared to the total height of the spine.
- X-ray criteria: lower limit of thoracic kyphosis below T12.

Statistics: sensitivity, specificity, positive (PPV) and negative predictive values (NPV), ROC curve.

**Results:**

FST showed a good reliability in detecting JK: with a threshold of 75 %, PPV was 100 %, NPV was 86 % and the Area Under the Curve was 83 %.

**Conclusion:**

The need for a useful criteria able to characterize JK to allow diagnosis and monitoring of the deformity is still lacking, and further studies will deepen this issue.

## Background

The human spine, in the sagittal profile, is organized in successive curvatures in which the lumbar lordosis plays a crucial role in the biomechanics and balance of the entire spine [1–3].

**Fig. 1:**
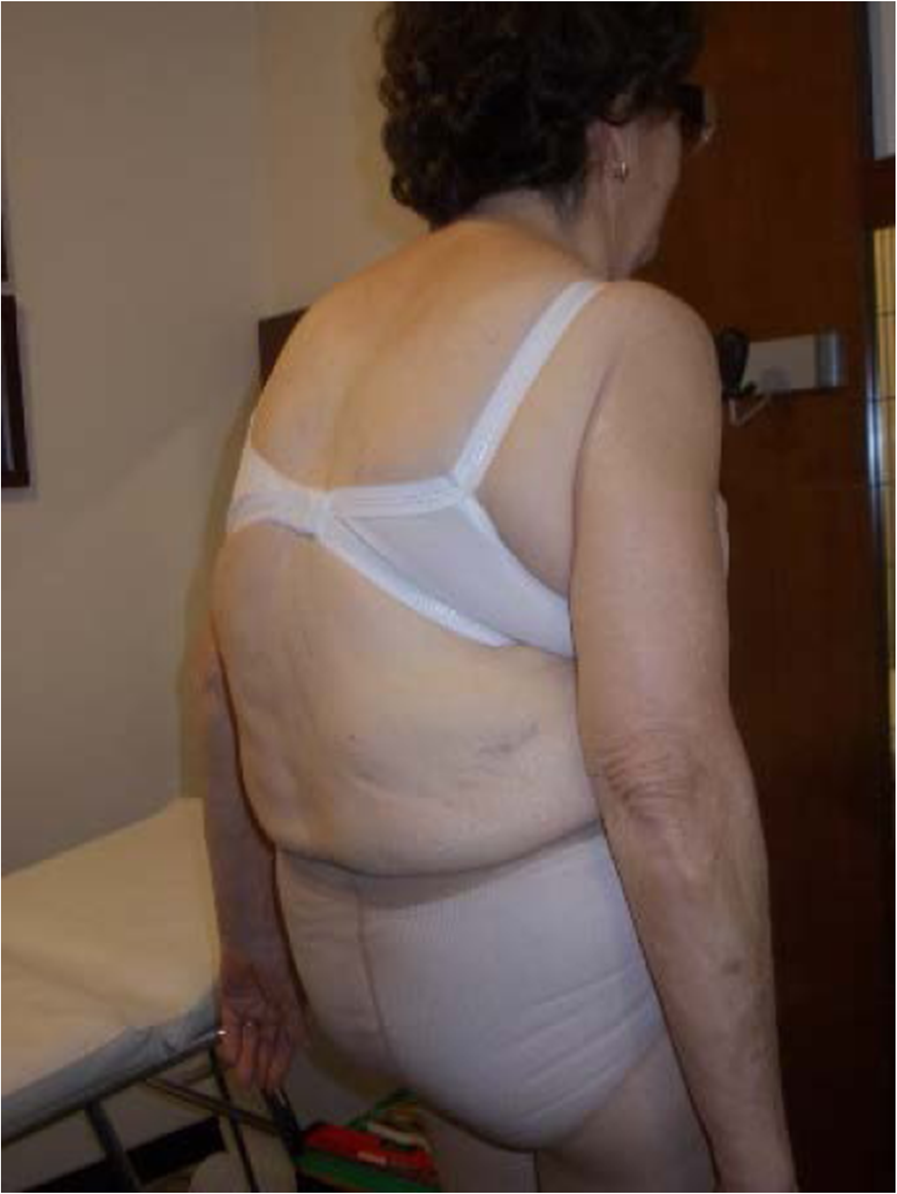
An old woman with thoraco-lumbar hyperkyphosis

The loss of lumbar lordosis, as well as the junctional kyphosis, are considered responsible for back pain and disability in adults with spine deformities [4–7] (Fig 1).

**Fig. 2:**
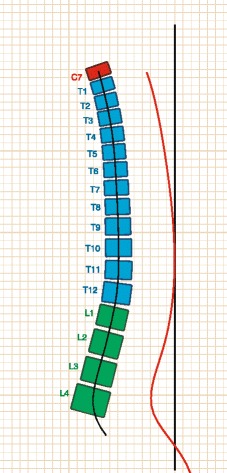
Formetric surface topography of a Junctional Kyphosis patient: inflection point is low and thoracic kyphosis includes some lumbar vertebrae

In healthy subjects, the thoraco-lumbar inflexion point is expected to be observed at T12, or at least at L1. If the inflexion point is below L1, the thoracic kyphosis includes some lumbar vertebrae (Fig. 2) and this is known as the “Junctional Kyphosis”. During growth this peculiar sagittal pattern can be associated with Scheuermann deformities, with scoliosis deformities and in some cases pain in adolescents.

Despite its importance in adult life, no study has ever evaluated its prevalence and evolution in growing patients. No universal criteria have been defined to diagnose and evaluate Junctional Kyphosis (JK) and the only available gold standard is the radiographic standard reference: JK occur when the thoracic kyphosis lower limit is at L1 or below. The actual gold standard is affected by the arms position, which modifies the sagittal spine posture and possibly also alters the inflexion level (8). Therefore, the radiographic standard reference may not be considered the best possible one. A clinical tool, like the sagittal index and the rastereographic evaluations showed to be reliable and feasible in detecting and monitoring hyperkyphosis [8, 9], but no tools exist to evaluate JK.

The aim of this study was to verify the sensitivity and specificity of Formetric evaluations in identifying Junctional Kyphosis patients in respect to the radiographical reference.

## Methods

Design: It is a cross sectional study, subjects came from a prospective database started in March 2003.

Participants: 22 patients provided by a Formetric evaluation at their first visit and a matchable lateral x-ray.

Comparisons: Formetric data of the group of patients with JK were compared to the formetric data of a group of 97 healthy subjects, retrieved from a screening program.

### Inclusion criteria

- JK diagnosis
- Availability of x-rays, in the frontal and lateral projection.
- Both idiopathic and Scheuermann JK were included.
- Formetric evaluation availability, within 1 month from the first visit.
- To create a homogeneous sample, both patients and control group subjects were selected by age according to Risser score 1 [10]. Inclusion criteria for males was age between 13 to 16; for females 12 to 14.

### Exclusion criteria

- Scoliosis diagnosis (Cobb over 10°)
- Other spine pathologies
- Spine surgery

### Diagnostic tests used to detect JK

**Fig. 3:**
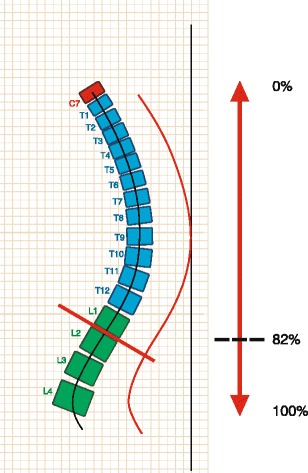
Formetric surface topography: level of inflexion in percentage compared to the total height of the spine

The level of thoraco-lumbar inflexion point in percentage compared to the total height of the spine, at the Formetric evaluations. In JK it was expected to be lower than in healthy subjects (Fig 3).

Statistics: Sensitivity, specificity, and Positive Predictive Value of each tool used to detect JK, was calculated using a 2×2 table. Positive Predictive Value was used to check the probability that in the case of a positive JK test, the patient really had the corresponding sagittal alteration. In all analyses.

*p* < 0.05 was considered statistically significant. Two Receiver Operating Characteristic (ROC) Curves were created to find the best threshold for both the plumbline distance and the percentage of the inflexion point.

## Results

After checking the lateral x-rays of the selected subjects, 22 had the lower limit of kyphosis below L1 and were assigned to the Junctional Kyphosis group (JK Group). 97 healthy subjects with a Formetric evaluation, who resulted negative at screening belonged to the control group.

Formetric surface topography showed a good reliability in detecting JK at the threshold of 75 %, with a Positive Predictive Value of 100 %.

**Fig. 4:**
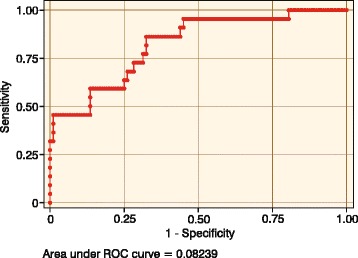
ROC curve of SFT 75 % threshold

For each parameter, to estimate optimal cut off values, a ROC curve was created (Fig. 4). The Area Under the Curve (AUC) was 83 %, which can be interpreted as the probability of correctly discriminating between healthy and JK patients.

## Discussion

This study showed that it is possible to detect JK with a Formetric evaluation, however there are some important limitations that must be taken into account to interpret the present findings. The sample size is small and it is unbalanced in respect to the control group, therefore there is the risk that the null hypothesis is confirmed by chance. However, we aimed to suggest some preliminary considerations for future analyses in larger groups of patients affected by Junctional Kyphosis.

Currently, evidence in this field is so scarce that it is difficult to compare the present results to other studies. Most of the current literature has mainly focused on how to treat thoracic hyperkyphosis and the relationship between sagittal imbalance and back pain in adulthood and old age. There is no evidence regarding how to properly identify and follow-up Junctional Kyphosis in children and adolescents through non-radiological means [11, 12].

**Fig. 5:**
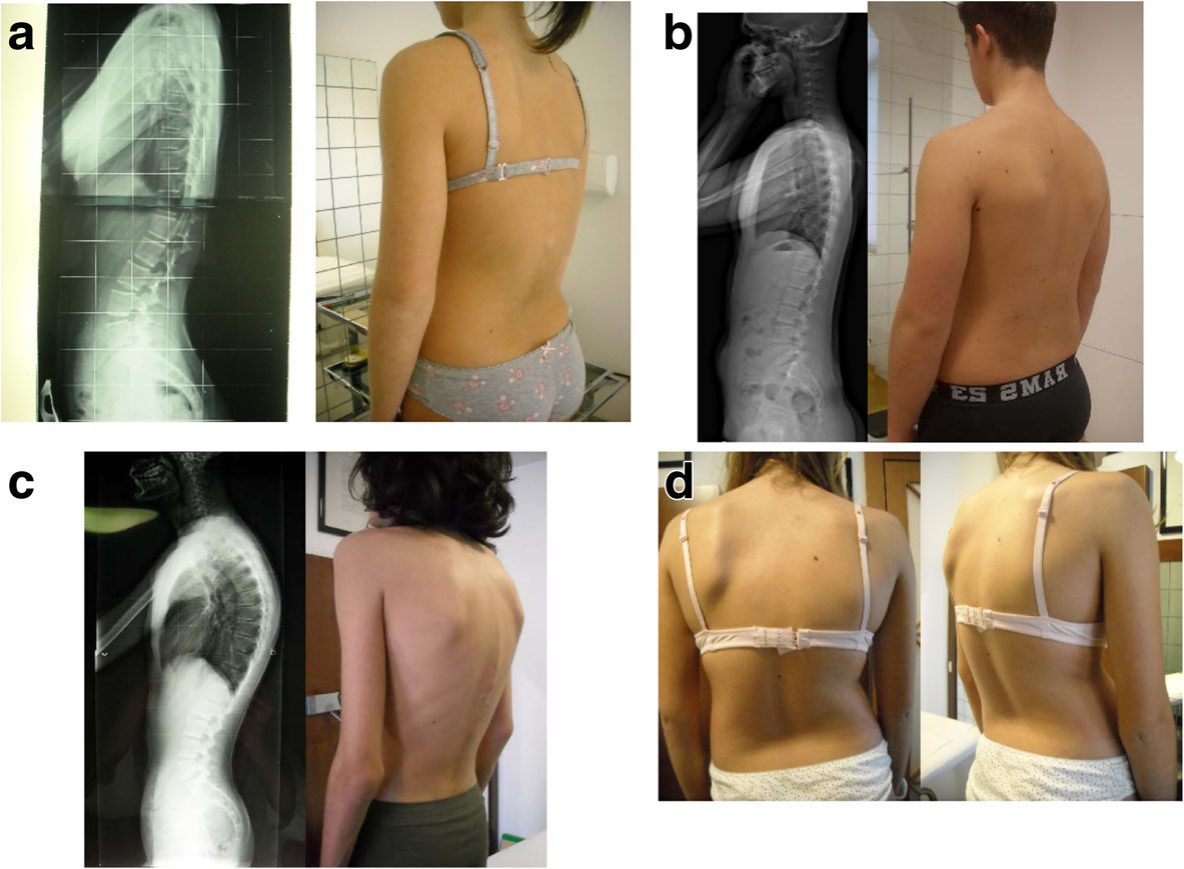
**a** Junctional kyphosis with reduced thoracic kyphosis; **b** Long armonic kyphosis; **c** Long thoracic hyperkyphosis; **d** Thoraco-lumbar scoliosis with Junctional Kyphosis
- A long armonic kyphosis (Fig. 5b)
- A long thoracic hyperkyphosis (Fig 5c)
- A thoraco-lumbar scoliosis with junctional kyphosis (Fig. 5d)

Junctional Kyphosis is a clinical condition not completely defined, and in fact it can be very hard to distinguish real disease from the healthy condition. In adolescents with Junctional Kyphosis, exercise treatment is prescribed to prevent pain and disability in adulthood, while bracing is prescribed only in the case of structural deformities or Scheuermann’s disease. Literature is not able to provide sufficient data to clearly define this group of spine disorders. There is an extreme variability of patterns:

- Junctional Kyphosis with reduced thoracic Kyphosis (Fig. 5a)

Even if Formetric seems to be able to detect JK among healthy subjects, it is not able to distinguish each subgroup of JK and it is not able to distinguish patients with Thoracic Hyperkyphosis from patients with JK. Before performing the current study, a preliminary analysis was done, to compare subjects with thoracic hyperkyphosis, with Junctional Kyphosis subjects.

Further studies are needed to better understand this large group of spine deformities. Future development will include research studies aiming to investigate a feasible and reliable tool, to diagnose and monitor Junctional Kyphosis during growth and in the elderly, to improve the understanding of the correlations of these deformities with pain and disability. Larger sample size and a better balance between the control and the affected groups, will guarantee a higher internal and external validity.

In addition, it is now clear that there is a need for a classification system to help clinical decision making and also to estimate the correlated risks for non-treated subjects.

## Conclusion

This study showed that it is possible to detect JK with a Formetric evaluation, but a useful criteria able to characterize JK to allow diagnosis and monitoring of the deformity is still lacking and further studies will help develop solutions for this issue.

## Abbreviations

JK: Junctional kyphosis
FST: Formetric surface topography
PPV: Positive predictive value
NPV: Negative predictive value
